# Survival Modeling Using Deep Learning, Machine Learning, and Statistical Methods: A Comparative Analysis for Predicting Mortality After Hospital Admission

**DOI:** 10.34133/hds.0449

**Published:** 2026-04-20

**Authors:** Ziwen Wang, Jin Wee Lee, Tanujit Chakraborty, Yilin Ning, Mingxuan Liu, Feng Xie, Marcus Eng Hock Ong, Nan Liu

**Affiliations:** ^1^ Centre for Biomedical Data Science, Duke-NUS Medical School, Singapore, Singapore.; ^2^ Department of Science and Engineering, Sorbonne University, Abu Dhabi, UAE.; ^3^ Division of Computational Health Sciences, Department of Surgery, University of Minnesota, Minneapolis, MN, USA.; ^4^ Programme in Health Services Research & Population Health, Duke-NUS Medical School, Singapore, Singapore.; ^5^ Department of Emergency Medicine, Singapore General Hospital, Singapore, Singapore.; ^6^ NUS Artificial Intelligence Institute, National University of Singapore, Singapore, Singapore.

## Abstract

**Background:** Survival analysis is essential for studying time-to-event outcomes and providing a dynamic understanding of the probability of an event occurring over time. Various survival analysis techniques, from traditional statistical models to state-of-the-art machine learning algorithms, support healthcare intervention and policy decisions. However, there remains ongoing discussion about their comparative performance. **Methods:** We conducted a comparative study of several survival analysis methods, including the accelerated failure time, Cox proportional hazards (CoxPH), stepwise CoxPH, elastic net penalized Cox model, random survival forests, gradient boosting machine learning, AutoScore-Survival, DeepSurv, time-dependent Cox model based on neural network, and DeepHit survival neural network. We applied the concordance index (C-index) for model discrimination, and the integrated Brier scores (IBSs) for calibration, and considered the model interpretability. The prediction performance was independently evaluated in the inpatient dataset of Singapore General Hospital (SGH) from 2017 to 2019 and Asian patients from the MIMIC-IV Clinical Database (MIMIC-IV). The outcome was to predict 90-d all-cause mortality based on patient demographics, clinicopathological features, and historical data. **Results:** The results of the C-index indicate that deep learning achieved comparable performance, with DeepSurv producing the best discrimination in both SGH (C-index: 0.893) and MIMIC-IV (C-index: 0.794). The calibration of DeepSurv also performed the best, with the IBS of 0.0406 in SGH and 0.1473 in MIMIC-IV, all using the full variables. Moreover, AutoScore-Survival, using a minimal variable subset, is easy to interpret and can achieve good discrimination (C-index in SGH: 0.867; MIMIC-IV: 0.788) and calibration (IBS in SGH: 0.0439; MIMIC-IV: 0.1263). **Conclusion:** All survival models were satisfactory in predicting mortality after hospital admission. This study provides recommendations for selection based on the characteristics of different models.

## Introduction

Survival analysis is a statistical field that focuses on studying the time-to-event of interest, such as death, recurrence, or failure [1]. It not only handles censored data effectively but also provides a more dynamic understanding of the probability of an event occurring over time [2]. Binary analysis alone is inadequate for dynamically capturing changes in states, such as qualitative transitions from alive to dead, while classical regression analysis cannot address the complexities associated with censoring [3]. Additionally, event times in medical studies often exhibit heavily skewed distributions, limiting the usefulness of statistical tests that assume a normal data distribution, even when there is no censoring in the dataset [4]. Therefore, the survival outcome differs from other outcomes in that it targets both time-to-event and censored status.

Historically, survival analysis has often relied on statistical models such as the Cox proportional hazards (CoxPH) model [5] and the accelerated failure time (AFT) model [6,7]. More recently, machine learning has made marked progress in the domain of survival analysis. Several promising machine learning algorithms for survival analysis have been developed, such as the random survival forest (RSF) [8] and DeepSurv [9]. Despite the considerable potential of applying machine learning in biomedical research and healthcare, concerns have emerged stemming from the lack of transparency in black-box models [10]. Thus, interpretable machine learning (IML) has emerged as a viable solution and gained prominence as an active area of research [11,12]. To gain a better understanding of the practical performance of diverse survival models and offer a valuable point of reference for clinical researchers uncertain about the actual performance of complex quantitative models, comparative studies utilizing real-world datasets are essential.

Regarding comparative studies of survival models, there is a vast amount of existing literature [13–16]. These studies have primarily focused on comparisons based on mathematical theory [13] and experimental comparisons using real-world data [14–16]. Currently, most comparative studies based on real medical data are disease-specific and lack comparison with IML models. Additionally, comparative studies using large-scale electronic health records (EHR) data are still primarily concentrated on nonsurvival models, while survival models based on EHR data require further exploration [17–19]. To fill this gap, we conducted a performance comparison of state-of-the-art algorithms under the survival framework to examine their strengths and weaknesses while also considering model interpretability. We sought to illustrate this by predicting all-cause mortality using real-world healthcare data from the emergency department (ED) of a large tertiary hospital and a publicly available MIMIC-IV Clinical Database (MIMIC-IV), respectively. First, we examined the differences between statistical modeling and machine learning based on the interpretability–performance trade-off, and provided comparisons of modeling processes. Second, in addition to considering continuous-time machine learning algorithms based on the proportional hazards (PH) assumption, we also explored machine learning methods under non-PH assumption or involving the discretization of time-to-event outcomes. Third, due to the presence of censoring in survival data, standard evaluation metrics for regression such as mean square error (MSE) and R-squared are not suitable for measuring the performance in survival analysis [2]. Most comparison studies for survival models typically focus on discriminative performance, such as the concordance index (C-index), with few studies evaluating measures of the accuracy of predicted survival functions and calibration, such as the integrated Brier score (IBS). Consequently, our study incorporates survival metrics such as C-index and IBS, which are essential for measuring the model’s goodness-of-fit, providing a comprehensive assessment of discrimination, and assisting in the evaluation of calibration and stability of the models.

Additionally, the application of survival models can support decision-makers in managing high-risk patients after admission based on their health characteristics. Key survival applications include dynamic risk evaluation (DRE; i.e., dynamically evaluating the risk of death), prediction of the risk period (PRP; i.e., assessing the time-to-event at a specified level of risk), and risk probability prediction (RPP; i.e., estimating the probability of death within a specific time horizon), among others. Therefore, the right choice of survival model plays a crucial role in its applications supporting managerial decision-makers.

Overall, this study aims to compare various survival analysis techniques, ranging from traditional statistical models to state-of-the-art machine learning algorithms. We sought to assess and illustrate the comparative performance of these models by real-world data for survival analysis on 90-d all-cause mortality after hospital admission, providing valuable evidence for researchers and clinicians in choosing appropriate methods.

## Materials and Methods

### Study design and setting

A retrospective cohort study was conducted on patients who visited the ED of Singapore General Hospital (SGH), a large tertiary hospital in Singapore. Every year, the SGH ED receives more than 120,000 visits and refers over 36,000 patients for inpatient admissions [20]. EHR data analyzed in this study were obtained from Singapore Health Services. The research received approval from the Centralized Institutional Review Board of Singapore Health Services, and a consent waiver was provided for the collection and analysis of EHR data due to the retrospective nature of the study. Additionally, all data underwent deidentification.

Another retrospective study comes from a comprehensive, freely accessible database, Medical Information Mart for Intensive Care IV (MIMIC-IV). The ED datasets generated and analyzed during the current study are available based on the study [21] or from the corresponding author upon reasonable request. The data have been de-identified to protect patient privacy while enabling research use.

### Study population

For the first retrospective cohort study, all patients hospitalized after visiting the ED of SGH between January 2017 and December 2019 were included. Each admission record of the patient was evaluated for multiple admission records for the same patient. The exclusion criteria were (a) patients younger than 21 years, (b) noncitizen patients who might not have complete medical records in the EHR system, and (c) suspected duplication of reports. Available variables included in the study were demographic information, vital signs, lab tests at baseline, comorbidities, and history. We extracted these candidate variables before hospital admission. Patients were excluded if any of the corresponding variables had more than 20% missing data [22]. For other missing variables, we filled in the data with the multiple imputation method by chained equations (MICE) [23] using predictive mean matching. In the study, the full dataset was randomly split into a non-overlapping training cohort (70%), validation cohort (10%), and test cohort (20%). For the second retrospective cohort study, all Asian patients over the age of 21 who were admitted to the intensive care units (ICU) after visiting the ED in the MIMIC-IV database were included, to align with the SGH cohort. Subsequently, patients with incomplete medical records and those suspected of having duplicate reports were also excluded.

### Data processing

The primary outcome of this study was all-cause mortality within 90 d for all hospitalized patients. Individuals that did not die within the designated 90-d period were considered right-censored, including those who were lost to follow-up or did not die during the study period.

Sixty preselected candidate variables in SGH and 25 candidate variables in MIMIC-IV were collected based on data availability, clinician opinion, and literature review [24]. The set of variables was classified into categorical and continuous variables since the preprocessing techniques are different for each type. For categorical variables such as race, triage class, malignancy, liver diseases, and diabetes, one-hot encoding was performed to convert the different categories into a binary feature. As continuous variables were the numerical input features, standardization is performed in the deep learning algorithms by subtracting the mean from each variable and dividing by the original standard deviation (SD) to support optimizing the model during training and to avoid getting stuck in local minima. However, it was found that the normalization of these variables in traditional survival models did not improve the performance or stability in any way. Comorbidities were obtained from hospital diagnoses and discharge records for patients’ index emergency admissions that occurred within the 5 years prior. All diagnoses were recorded using International Classification of Diseases (ICD) codes (ICD-9/ICD-10) [25], a globally adopted diagnostic tool for epidemiological and clinical purposes. The comorbidity variables were extracted using the Charlson comorbidity index (CCI) [26], and the algorithms proposed by Quan et al. [27] were applied in this study to link the CCI with the ICD codes. The list of candidate variables and abbreviations were given in Table S1.

### Statistical analysis

**Fig. 1.**
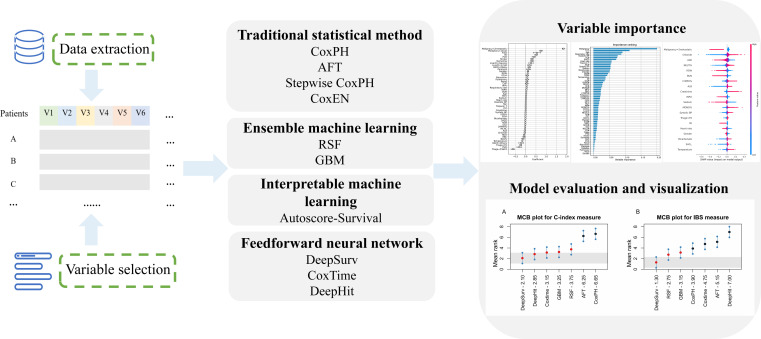
Flowcharts of the study design.

We examined the baseline characteristics of the study population for the training, validation, and test cohorts. In the descriptive summary, we tested the candidate variables under different event statuses. The research process is illustrated in Fig. 1, including variable selection, data extraction, model development, model validation, and evaluation.

We selected 10 models that are representative in survival analysis and have been widely used in clinical studies, including 4 that are based on the traditional survival methods [AFT model [6,7], CoxPH model [5], the stepwise CoxPH model [28,29], and the elastic net penalty Cox model (CoxEN) [30,31]], 3 models from machine learning paradigm [AutoScore-Survival [12], RSF [8], and gradient boosting (GBM) [32]], and 3 deep neural network algorithms [DeepSurv [9], time-dependent Cox models (CoxTime) [33], and DeepHit [34]]. To better understand the assumptions, interpretability, and the necessity for parameter tuning in different methods, these methods are described in Table 1.

**Table 1.** Description of various methods

Classification	Models	Proportional hazards assumption	Interpretability	Parameter tuning
Traditional statistical method	AFT model	No	High	No
	CoxPH model	Yes	High	No
	Stepwise CoxPH	Yes	High	No
	CoxEN	Yes	High	Yes
Ensemble machine learning	RSF	No	Moderate	Yes
	GBM	No	Moderate	Yes
Interpretability machine learning	AutoScore-Survival	Yes	High	Yes
Feedforward deep neural network	DeepSurv	Yes	Low	Yes
	CoxTime	No	Low	Yes
	DeepHit	No	Low	Yes

We fitted models using a training cohort, fine-tuned the parameters based on the validation cohort to achieve optimal performance, and ultimately assessed the performance based on the test cohort. In some traditional models such as CoxPH, which do not need parameter tuning, the validation set does not play any role in the modeling phase. The univariate and multivariate CoxPH regression were applied to identify clinically relevant predictors.

#### Variable selection strategy

Among the algorithms considered, a key comparison involves the simultaneous conduct of variable selection and estimation, with 2 traditional methods (stepwise CoxPH and CoxEN) and 3 machine learning methods (RSF, GBM, and AutoScore-Survival).

Stepwise CoxPH: Forward and backward selection were conducted using the Akaike information criterion (AIC) to balance model fit and parsimony.

CoxEN model: Simultaneous variable selection and coefficient estimation were performed via penalized partial likelihood. The penalty parameters α and λ were tuned using 5-fold cross-validation optimized on the C-index, achieving the optimal trade-off between bias and variance.

RSF and GBM: Both ensemble methods were evaluated under full-variable and reduced-variable settings. Variable importance was computed based on mean decrease in prediction error (RSF) or average gain (GBM). The number of selected variables was determined by balancing model performance, measured by C-index, and model complexity, represented by the number of predictors, using an elbow method to identify the point beyond which additional variables led to negligible improvement in C-index.

AutoScore-Survival: Variables were ranked by importance scores derived from RSF in the initial step. A parsimony plot was used to determine the optimal number of variables based on validation cohort performance. AutoScore generates interpretable risk scores, which were subsequently transformed into mortality probabilities via the Nelson–Aalen estimator to obtain individualized survival predictions.

#### Hyperparameter optimization

For the ensemble machine learning RSF and GBM, a grid search based on 5-fold cross validation was used during training to tune hyperparameters. For 3 deep neural network algorithms, the hyperparameters include learning rate, hidden layers, nodes per layer, dropout, and batch size, a set of hyperparameters was set for tuning to achieve optimal performance, as determined by the iterative training and validation cycles. Early stopping regularization was also applied to the deep learning-based algorithms to stop model training when no further improvement in performance on the validation cohort was observed. The hyperparameter tuning set and software used for all techniques are shown in Table S4.

### Evaluation criteria for survival prediction

Specialized performance evaluation metrics for survival analysis were employed, including Harrell’s C-index and IBS [35]. Detailed mathematical definitions can be found in the Supplementary Materials.

C-index: The C-index is a standard performance measure in survival analysis, which can be defined as the proportion of concordance pairs in a population [35–37]. It was originally introduced by Frank E. Harrell [36,37] as a time-independent performance measure, with values ranging from 0 to 1, where a value of 1 indicates perfect performance and 0 represents the worst possible performance. If a model makes random predictions without considering any information from the data, the corresponding C-index would be around 0.5. In practice, a higher C-index reflects better model discrimination [14].

IBS: The Brier score (BS) [38] is frequently utilized to quantify the mean square difference between the observed patient status and the predicted survival probabilities at a particular point in time. To some extent, BS is similar to MSE for regression models, but it is time-dependent due to the dynamic status and employs the inverse probability of censoring weights to deal with censored subjects [39]. For global interpretability, the IBS can be calculated, as it does not consider specific time points but rather takes all available time points as a whole. IBS possesses the appealing feature of simultaneously accounting for discrimination and calibration [40]. It typically ranges from 0 to 1, representing perfect and worst discrimination and calibration, respectively. In practice, a model with IBS below 0.25 is deemed useful [14–16].

## Results

### Patient characteristics

**Fig. 2.**
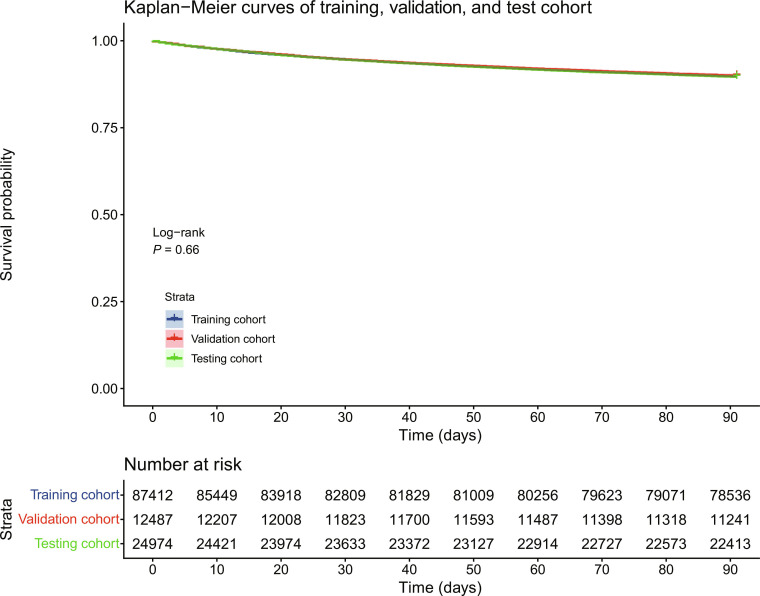
Kaplan–Meier curve of training and testing cohorts in SGH. There was no statistically significant difference between the survival of training and testing cohorts in the log-rank test (*P* = 0.66).

We conducted a statistical description of the baseline characteristics of the study cohort, helping to identify key factors that could influence the 90-d all-cause mortality prediction, thereby improving the predictive accuracy of the models. Sixty variables in SGH, routinely available for patients admitted to the hospital from ED, were included in this study, consisting of 43 continuous variables and 17 categorical variables (see Table S1). A total of 124,873 inpatient admission episodes were finally included, with 87,412 episodes in the training cohort, 12,487 episodes in the validation cohort, and 24,974 in the testing cohort. The Kaplan–Meier curve for the overall population was plotted in Fig. 2. Among the included episodes, 112,118 (89.8%) survived for more than 90 d when censored at the end of the designated 90-d observation period. In contrast, 12,755 (10.2%) episodes had a death event within 90 d, with a median time to death of 28 d [interquartile range (IQR): 11 to 53] and a mean time to death of 34 d (SD: 25.7). Table S2 summarizes the candidate variables under different event statuses. Continuous variables were presented as either mean and SD or median and IQR. Student’s *t* tests were applied to test continuous variables that followed normal distributions, while the Mann–Whitney *U* test was used for non-normal continuous variables. Categoric variables were expressed as counts and percentages, and tested using either chi-square or Fisher exact tests. There were statistically significant differences in the features between different event status. Without loss of generality, we primarily reported the results from the SGH data, with the corresponding MIMIC-IV results included in Tables S3 and S5 and Fig. S1.

### Variable selection and importance

We reported the results of variable selection and provided the variable importance to explore the model interpretability. Table 2 presents the results of the univariate and multivariate analyses of the Cox model for all variables. From univariate analysis, it is observed that basophil absolute count, the total blood cell count, rheumatic, paralysis, and diabetes without chronic complications did not exhibit statistical significance at the 0.001 level. All variables other than these can be considered risk factors for inpatients. As a result, selecting a parsimonious model based only on P value is challenging. The effects based on AFT model are shown in Fig. S2.

**Table 2.** Univariate and multivariate analysis of the Cox model in SGH for survival probability

Variables	Univariate Cox regression		Multivariate Cox regression	
	Hazard ratio (95%)	*P* value	Hazard ratio (95%)	*P* value
Age (years)	1.034 (1.032–1.035)	<0.001	1.033 (1.031–1.035)	<0.001
Gender (male)	1.202 (1.153–1.253)	<0.001	1.151 (1.102–1.202)	<0.001
Race				
Chinese	-	-	-	-
Indian	0.578 (0.532–0.629)	<0.001	0.990 (0.908–1.079)	0.8140
Malay	0.747 (0.697–0.802)	<0.001	1.260 (1.172–1.356)	<0.001
Others	0.539 (0.471–0.617)	<0.001	0.804 (0.701–0.921)	0.0016
Triage class				
P1	-	-	-	-
P2	0.509 (0.487–0.531)	<0.001	0.763 (0.728–0.801)	<0.001
P3&P4	0.174 (0.157–0.194)	<0.001	0.468 (0.419–0.524)	<0.001
Diastolic BP	0.974 (0.972–0.975)	<0.001	1.006 (1.004–1.008)	<0.001
Systolic BP	0.983 (0.983–0.984)	<0.001	0.993 (0.992–0.994)	<0.001
FIO_2_	3.759 (3.148–4.488)	<0.001	1.661 (1.383–1.994)	<0.001
Heart rate	1.020 (1.019–1.021)	<0.001	1.008 (1.006–1.009)	<0.001
Respiration rate	1.119 (1.112–1.125)	<0.001	1.044 (1.036–1.052)	<0.001
SAO_2_	0.973 (0.971–0.975)	<0.001	0.986 (0.984–0.989)	<0.001
Temperature	0.937 (0.913–0.962)	<0.001	0.843 (0.822–0.866)	<0.001
Blood albumin	0.868 (0.865–0.871)	<0.001	0.952 (0.948–0.956)	<0.001
Basophil absolute count	0.981 (0.866–1.112)	0.7685	1.018 (0.913–1.136)	0.7443
Basophil cell	0.360 (0.329–0.394)	<0.001	0.834 (0.769–0.904)	<0.001
Bicarbonate	0.953 (0.948–0.958)	<0.001	0.979 (0.972–0.985)	<0.001
Chloride	0.929 (0.926–0.932)	<0.001	0.935 (0.929–0.941)	<0.001
Serum creatinine	1.000 (1.000–1.001)	<0.001	0.999 (0.999–0.999)	<0.001
Eosinophil absolute count	0.787 (0.716–0.865)	<0.001	1.049 (1.014–1.084)	0.0055
Eosinophil cell	0.897 (0.886–0.907)	<0.001	0.970 (0.959–0.981)	<0.001
Blood glucose	1.009 (1.005–1.013)	<0.001	0.982 (0.977–0.987)	<0.001
Hematocrit	0.918 (0.915–0.920)	<0.001	1.252 (1.193–1.314)	<0.001
Hemoglobin	0.783 (0.777–0.790)	<0.001	0.564 (0.497–0.641)	<0.001
Lymphocyte absolute count	0.624 (0.605–0.643)	<0.001	1.014 (1.005–1.023)	<0.001
Lymphocytes cell	0.935 (0.933–0.938)	<0.001	0.956 (0.951–0.959)	0.0030
MCHB	1.023 (1.016–1.030)	<0.001	0.776 (0.684–0.881)	<0.001
MCHC	0.872 (0.862–0.884)	<0.001	1.440 (1.282–1.617)	<0.001
Mean corpuscular volume	1.028 (1.025–1.031)	<0.001	1.087 (1.042–1.133)	<0.001
Mean platelet volume	1.057 (1.035–1.080)	<0.001	1.051 (1.028–1.075)	<0.001
Monocyte absolute	1.036 (1.030–1.043)	<0.001	1.008 (0.991–1.026)	0.3494
Monocyte cell	0.980 (0.974–0.987)	<0.001	0.975 (0.967–0.982)	<0.001
Neutrophil absolute count	1.026 (1.025–1.027)	<0.001	1.003 (0.998–1.008)	0.2744
Neutrophil cell	1.042 (1.040–1.044)	<0.001	0.978 (0.974–0.982)	<0.001
Platelet count	1.000 (1.000–1.001)	<0.001	1.000 (0.999–1.000)	0.0281
Serum potassium	1.328 (1.289–1.368)	<0.001	1.098 (1.063–1.135)	<0.001
Red blood cell	0.476 (0.465–0.488)	<0.001	0.632 (0.517–0.773)	<0.001
Red cell distribution width	1.214 (1.208–1.220)	<0.001	1.107 (1.098–1.117)	<0.001
Serum sodium	0.937 (0.934–0.940)	<0.001	1.031 (1.023–1.038)	<0.001
Total absolute count	1.008 (1.008–1.009)	<0.001	1.009 (0.972–1.047)	0.6382
Total blood cell count	1.373 (1.085–1.738)	0.0083	1.280 (1.019–1.608)	0.0339
Troponin T	1.000 (1.000–1.000)	<0.001	1.000 (1.000–1.000)	0.0514
Blood urea nitrogen	1.045 (1.043–1.047)	<0.001	1.029 (1.026–1.032)	<0.001
White blood cell	1.008 (1.008–1.009)	<0.001	0.987 (0.952–1.024)	0.4982
MI	2.894 (2.714–3.086)	<0.001	1.664 (1.551–1.785)	<0.001
CHF	1.766 (1.655–1.855)	<0.001	1.023 (0.954–1.098)	0.5210
PVD	1.728 (1.591–1.877)	<0.001	1.533 (1.405–1.672)	<0.001
Stroke	1.125 (1.052–1.202)	<0.001	1.409 (1.309–1.517)	<0.001
Dementia	1.765 (1.612–1.933)	<0.001	1.301 (1.183–1.431)	<0.001
Pulmonary	1.213 (1.127–1.306)	<0.001	1.068 (0.988–1.154)	0.0999
Rheumatic	0.751 (0.605–0.932)	0.0094	0.989 (0.796–1.230)	0.9226
PUD	1.619 (1.435–1.827)	<0.001	0.685 (0.605–0.776)	<0.001
Paralysis	1.180 (1.066–1.306)	0.0014	1.144 (1.025–1.278)	0.0169
Renal	1.545 (1.477–1.616)	<0.001	1.074 (1.011–1.141)	0.0215
Malignancy				
None	-			
Local tumor leukemia and lymphoma	3.319 (3.112–3.539)	<0.001	2.205 (2.061–2.360)	<0.001
Metastatic solid tumor	10.526 (10.064–11.009)	<0.001	6.689 (6.343–7.055)	<0.001
LiverD				
None	-			
Mild	1.535 (1.393–1.691)	<0.001	1.255 (1.136–1.387)	<0.001
Severe	2.955 (2.655–3.289)	<0.001	1.372 (1.222–1.540)	<0.001
Diabetes				
None	-			
Diabetes without chronic complications	0.895 (0.782–1.024)	0.1062	0.981 (0.855–1.126)	0.7852
Diabetes with complications	1.262 (1.209–1.317)	<0.001	0.989 (0.940–1.041)	0.6820
ED#	1.032 (1.028–1.036)	<0.001	0.977 (0.959–0.997)	0.0212
INP#	1.081 (1.075–1.086)	<0.001	1.053 (1.028–1.079)	<0.001
SURG#	1.180 (1.165–1.196)	<0.001	1.012 (0.991–1.033)	0.2698
HD#	1.244 (1.205–1.285)	<0.001	0.902 (0.868–0.939)	<0.001
ICU#	1.421 (1.342–1.505)	<0.001	1.084 (1.011–1.162)	0.0227

After variable selection, the stepwise CoxPH retained 50 out of the total 60 variables. Figure S3 shows the 50 coefficients of all retained variables in the stepwise CoxPH model, ranked by log relative hazards. CoxEN retained 26 variables, and the estimated shrunken coefficients for all the retained variables are presented in Fig. S4. For AutoScore-Survival, 16 variables were selected based on the balance between model performance measured by the C-index and the complexity represented by the number of variables. The 16-variable survival score is tabulated in Table S6, taking into account malignancy, total cell count, age, respiratory rate, diastolic blood pressure (BP), albumin, SAO_2_, heart rate, troponin T quantitative, blood urea nitrogen, systolic BP, sodium, chloride, basophil absolute count, and red cell distribution width. The above models offer a sufficient level of interpretability, enabling clinicians to understand the impact of variables on mortality through coefficients or scores directly.

For RSF, the variable importance is consistent with that obtained in step one of AutoScore-Survival, shown in Fig. S5. Additionally, Fig. S6 presents the variable importance for the GBM method. It is evident that the variable importance from RSF and GBM is not as intuitive and interpretable as scores, as it only provides the overall contribution. For the deep neural network algorithms (DeepSurv, CoxTime, and DeepHit), all variables were used for training. To provide insights into the predictions made by the deep neural network, we incorporated SHapley Additive exPlanations (SHAP) analysis to aid in model interpretability (Figs. S7 to S9). The results showed that malignancy, specifically metastatic solid tumors, was the variable with the greatest impact on survival time, which is consistent with the most important variables identified by other models.

### Performance comparisons

To compare the performance of all the prediction algorithms on the SGH and MIMIC-IV dataset, we report in Table 3 the mean, standard error (SE), and 95% confidence interval (CI) of the C-index and IBS for all algorithms using the same testing cohort that was completely isolated from the training cohort. Additionally, Table S8 summarizes the secondary attributes of each model to guide model selection in real-world applications.

**Table 3.** Performance of different methods with/without variable selection mechanisms

	SGH			MIMIC-IV		
		Evaluation criteria			Evaluation criteria	
Methods	No. of variables	C-index (SE)	CI (95%)	No. of variables	C-index (SE)	CI (95%)
AFT	60	0.880 (0.0030)	0.874–0.885	25	0.785 (0.0632)	0.674–0.900
CoxPH	60	0.879 (0.0031)	0.873–0.885	25	0.784 (0.0629)	0.671–0.900
CoxEN	26	0.875 (0.0035)	0.868–0.882	24	0.770 (0.0731)	0.607–0.900
Stepwise CoxPH	50	0.879 (0.0033)	0.872–0.886	7	0.778 (0.0572)	0.656–0.868
AutoScore -Survival	16	0.867 (0.0031)	0.861–0.873	7	0.788 (0.0430)	0.699–0.884
RSF	16	0.876 (0.0032)	0.871–0.882	7	0.736 (0.0456)	0.632–0.884
RSF	60	0.889 (0.0028)	0.883–0.895	25	0.772 (0.0529)	0.667–0.868
GBM	16	0.880 (0.0028)	0.874–0.885	7	0.783 (0.0318)	0.714–0.847
GBM	60	0.891 (0.0034)	0.884–0.898	25	0.786 (0.0410)	0.708–0.880
DeepSurv	60	0.893 (0.0032)	0.886–0.899	25	0.794 (0.0617)	0.661–0.905
CoxTime	60	0.891 (0.0027)	0.886–0.896	25	0.789 (0.0446)	0.685–0.870
DeepHit	60	0.892 (0.0031)	0.886–0.898	25	0.776 (0.0578)	0.658–0.861
Methods	No. of variables	IBS (SE)	CI (95%)	No. of variables	IBS (SE)	CI (95%)
AFT	60	0.0431 (0.0008)	0.0413–0.0444	25	0.3152 (0.5807)	0.0568–1.6134
CoxPH	60	0.0428 (0.0008)	0.0414–0.0443	25	0. 3371 (0.6356)	0.0532–1.7384
CoxEN	26	0.0445 (0.0010)	0.0426–0.0467	24	0.1725 (0.0397)	0.1120–0.2569
Stepwise CoxPH	50	0.0436 (0.0009)	0.0416–0.0457	7	0.2434 (0.0668)	0.1368–0.3740
AutoScore -Survival	16	0.0439 (0.0008)	0.0425–0.0456	7	0.1263 (0.0330)	0.0453–0.2275
RSF	16	0.0425 (0.0008)	0.0411–0.0440	7	0.1932 (0.0437)	0.1189–0.2780
RSF	60	0.0418 (0.0008)	0.0405–0.0434	25	0.1817 (0.0428)	0.1088–0.2717
GBM	16	0.0445 (0.0008)	0.0427–0.0459	7	0.1529 (0.0318)	0.0871–0.2173
GBM	60	0.0421 (0.0010)	0.0406–0.0442	25	0.1493 (0.0306)	0.1014–0.2034
DeepSurv	60	0.0406 (0.0009)	0.0390–0.0423	25	0.1473 (0.0307)	0.0949–0.2101
CoxTime	60	0.0429 (0.0008)	0.0412–0.0443	25	0.1568 (0.0420)	0.0883–0.2677
DeepHit	60	0.0489 (0.0010)	0.0470–0.0511	25	0.1765 (0.0313)	0.1234–0.2467

Table 3 shows the performance results of the C-index. Compared with the standard CoxPH model (C-index: 0.879) in SGH, 3 deep neural network algorithms showed better discrimination of all-cause mortality in hospitalized patients (C-index of DeepSurv: 0.893; CoxTime: 0.892; DeepHit: 0.891), with DeepSurv having the highest C-index of 0.893. Similarly, DeepSurv (C-index: 0.794) and CoxTime (C-index: 0.789) still maintained the best discrimination in MIMIC-IV. However, the above deep neural network algorithms used full candidate variables and lacked interpretability, which may hinder their clinical applicability.

The IBS in Table 3 showed that the consistency between the model prediction and the actual observation using all variables for all-cause mortality within 90 d was best for the DeepSurv model, in both SGH (IBS: 0.0406) and MIMIC-IV (IBS: 0.1473). Secondly, RSF and GBM demonstrated good predictive performance in SGH and MIMIC-IV, respectively.

When variable selection was applied, performance differed across datasets. In the SGH dataset, GBM demonstrated the highest discriminative performance (C-index: 0.880), while RSF achieved the best calibration (IBS: 0.0425), both reducing the number of variables from 60 to16. For the MIMIC-IV dataset, AutoScore-Survival reduced the number of variables from 25 to 7, achieving the best discrimination (C-index: 0.788) and calibration (IBS: 0.1263).

### Model visualization

**Fig. 3.**
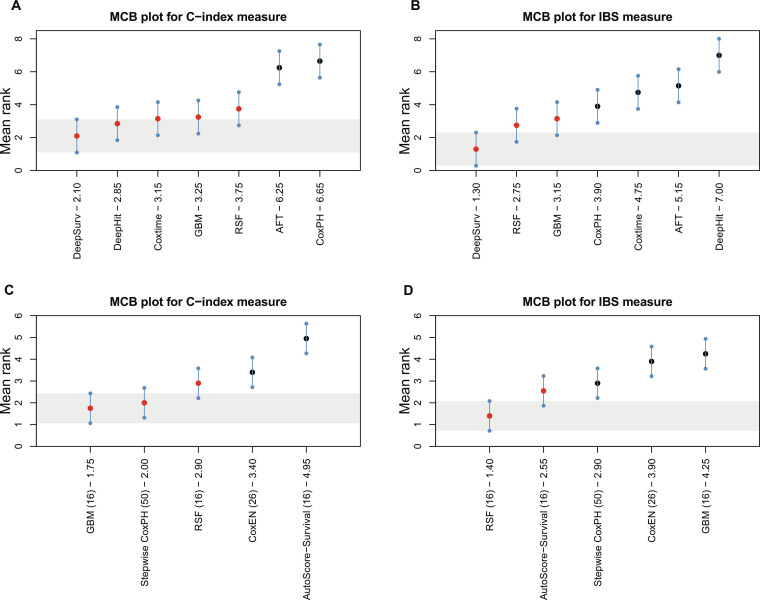
Visualization of the MCB test in terms of C-index (left) and IBS (right) measures based on SGH. (A and B) Models using full variables. (C and D) Models with variable selection. The dot in the middle is the mean rank of the method, where GBM (16)-1.45 indicates the mean rank as 1.45 for GBM, which selected 16 variables, and the line above and below the dot is the critical distance. Models whose confidence intervals overlap with this reference region are not statistically different from the best model at the specified significance level. The red dots indicate that the performance difference is insignificant, and the black dots represent a significant difference.

**Fig. 4.**
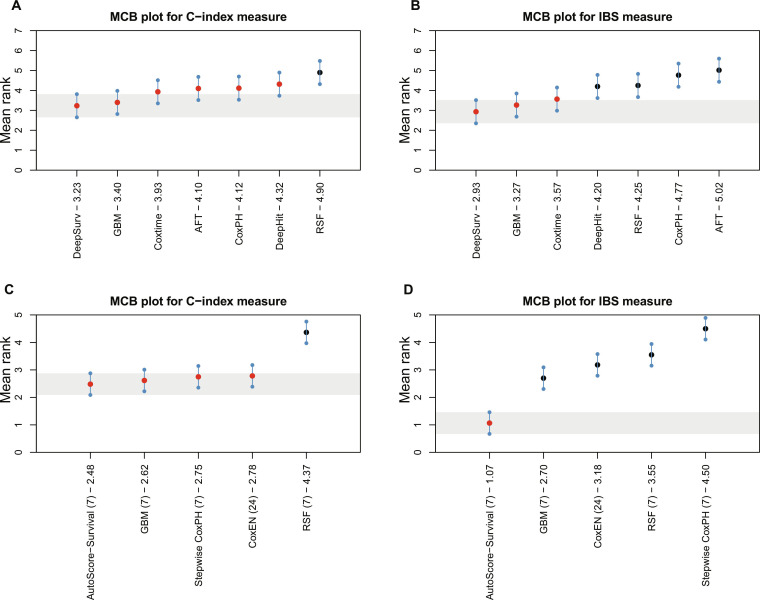
Visualization of the MCB test in terms of C-index (left) and IBS (right) measures based on MIMIC-IV. (A and B) Models using full variables. (C and D) Models with variable selection. Models whose confidence intervals overlap with this reference region are not statistically different from the best model at the specified significance level. The red dots indicate that the performance difference is insignificant, and the black dots represent a significant difference.

To assess the statistical significance of different models’ performance, we further conducted the Multiple Comparisons with the Best (MCB) test [41] for C-index and IBS measures. The MCB plot was developed to realize the visualization of the model, which added great clinical application value in choosing an appropriate model. As shown in Fig. 3 based on SGH and Fig. 4 based on MIMIC-IV, panels (A) and (B) correspond to models using all variables, while panels (C) and (D) correspond to models with variable selection. We simulate 20 observations for each method with specific mean and CIs. This nonparametric test calculates average ranks and critical distances for performance measures. For models using all variables, the MCB results reveal that DeepSurv performs best in terms of C-index and IBS measures, in both SGH and MIMIC-IV. For models using variable selection, Fig. 3 based on SGH shows that GBM exhibits the best discriminative ability, while RSF demonstrates robust calibration. However, in Fig. 4 based on MIMIC-IV, it is observed that AutoScore-Survival exhibits the best discrimination and calibration. The critical region of the best-performing model (shaded region) represents the reference value for the test. Models with critical regions overlapping the reference value do not demonstrate statistically significant performance differences, while models with the critical region above this value significantly underperform compared to the best-performing one. For example, in Fig. 3C, the substantial overlap between the GBM model and the stepwise CoxPH model indicates no statistically significant difference in discrimination, despite differences in nominal ranking. This finding underscores that simpler and more interpretable statistical models can remain competitive against complex machine learning models.

## Discussion

As advancements in data collection techniques continue to generate larger clinical datasets, it is crucial to identify the best methods for analyzing complex survival data. In this study, we provide a comprehensive benchmark evaluation of deep learning, several machine learning models, and traditional statistical models for survival analysis on 90 d of all-cause mortality after hospital admission using data from ED admissions at SGH and MIMIC-IV. In the application of survival models, model discrimination is crucial for DRE, and important for PRP and RPP to account for the differences in patient characteristics. Model calibration is essential for PRP and RPP to ensure the reliability of the underlying risk predictions.

Overall, the results demonstrate that a range of models are suitable for these applications, both well-calibrated and with relatively high discrimination. Across both bases for the ranking measure, the performance on each dataset varied slightly within a tight band. Applications of survival models are possible to support decision-makers in the management of patients at risk after hospital admission based on their health characteristics. Therefore, medical researchers and clinicians can choose suitable models based on varying requirements.

First, DeepSurv consistently demonstrated the best overall performance in both discrimination (C-index) and calibration (IBS) across datasets, highlighting its suitability for modeling complex nonlinear relationships in high-dimensional clinical data. Secondly, traditional survival models such as CoxPH and AFT can infer the effects of variables on survival time, providing a source of interpretability for survival prediction. Nevertheless, addressing the complexity of real-world data brings challenges for traditional survival models, such as the potential for infeasible fits [42]. In machine learning, more flexible optimization methods are commonly employed, which reduces the occurrence of such situations [43]. In addition, statistical models typically require an iterative development process, which often involves transforming highly skewed variables, examining residual plots to identify the need for nonlinear effects or interactions, and so on. Therefore, statistical modeling requires more human intervention and expertise, and it is more applicable for practitioners with statistical backgrounds.

Regarding interpretability, the increasing adoption of model-agnostic interpretability methods, such as SHAP and local interpretable model-agnostic explanations, has made even complex “black-box” models more interpretable and clinically actionable, thus bridging the gap between performance and transparency. AutoScore-Survival, while not outperforming deep learning models in discrimination, offers a highly interpretable and parsimonious alternative with strong calibration and transparent variable contribution, particularly valuable in resource-limited settings. Since the importance of each scoring variable is transparent, clinicians are more easily able to understand and trust the model outputs, contributing to the effectiveness and acceptability of such interpretable methods in real-world decision-making in medical and health applications.

Another crucial component of model development and comparison is variable selection, which is particularly important when dealing with clinical data due to the sheer number of patient variables at hand. Obtaining the most useful and parsimonious set of variables improves model coherence and contributes to a better understanding of the primary risk factors associated with all-cause mortality for inpatients. We compared 3 different approaches for variable selection. One common approach is the stepwise method, which calculates the AIC or bayesian information criterion by adding and/or eliminating variables to find the optimal model. A popular alternative to the stepwise method is the elastic net penalty method. Unlike the stepwise approach, the elastic net penalty method includes all variables in the model but shrinks some regression coefficients to zero. The third approach is based on RSF variable importance ranking, which is the first step of the AutoScore-Survival estimation process. We observed that all methods conclude that malignancy is the most important variable and has a significant impact on survival time. Furthermore, AutoScore-Survival identified age, SAO₂, malignancy, and respiratory rate as key risk-driving variables in both SGH and MIMIC-IV datasets, effectively capturing clinically meaningful factors associated with 90-d mortality after hospital admission. These variables provide clinicians with clear insights into why a particular patient is at high risk, guiding triage strategies, risk stratification, and resource allocation in clinical practice.

This study has several limitations. First, since the data were collected retrospectively, potential confounding factors may exist, which could influence both the exposure and the outcome. Additionally, institutional and regional variations, including discrepancies in data acquisition protocols, laboratory measurement standards, and coding systems, could also contribute to heterogeneity in model performance and partially explain the differences observed between datasets. Note that the MIMIC-IV database was used for analysis restricted to Asian patients to align baseline characteristics with the SGH cohort; therefore, the results reflect performance within a specific demographic subgroup rather than a fully heterogeneous population. Therefore, the performance of different methods may vary in different healthcare settings. These dataset-specific constraints may limit the generalizability of the findings across healthcare institutions. However, the process workflows for various models presented in this study can be generalized to other clinical datasets. Future studies should include prospective external validation on more demographically heterogeneous datasets and explore domain adaptation or federated learning strategies to enhance cross-institutional applicability.

Second, the benchmark dataset used in this study is based on large-scale EHR data, including routinely collected variables. However, certain features were not taken into consideration, for example, the Glasgow coma scale (GCS) score is excluded due to a high missing rate of 78%. GCS is considered an important predictive factor in an ED setting and could potentially improve the performance of these survival models.

Another limitation lies in the class imbalance of the outcome variable (high censoring). This imbalance could potentially bias model training and evaluation. Future research could incorporate imbalance correction techniques—such as resampling, or synthetic data augmentation—to examine their effect on model robustness and predictive accuracy. Although high censoring can increase prediction uncertainty, the large sample size and substantial number of observed events provided sufficient information for modeling in our study, and this effect can be mitigated. The improved performance of DeepSurv observed in our study is more plausibly attributable to its ability to model nonlinear effects and complex interactions.

In addition, further research could explore other types of statistical models or machine learning approaches, such as semi-supervised [44,45] and unsupervised learning [46] methods, to evaluate their performance on clinical datasets. More recently, attention-based deep learning models designed for tabular data, including Transformer-based survival architectures, have shown increasing promise in EHR research and may further improve predictive performance. Although such models were not included in the current comparative analysis, their rapid methodological development warrants systematic evaluation in future benchmark studies. Future work could also benefit from sensitivity analyses to examine robustness across different patient subgroups and healthcare environments. Considering that D-calibration [2] is a calibration measure, we can also regard D-calibration as a potential tool for future research. Despite these limitations, this study provides valuable evidence for researchers in choosing the most appropriate method.

## Conclusion

This study sought to present a comprehensive comparison of various survival analysis techniques for predicting all-cause mortality after hospital admission based on the interpretability–performance trade-off. Overall, all methods demonstrated satisfactory performance, although the ranking of performance for each dataset varied slightly within a narrow range. However, machine learning and statistical modeling differ in their workflow processes. It is important to select a suitable method for specific data analysis and application requirements. Black-box machine learning models demonstrate strong discrimination and predictive performance but require external tools for model interpretability. Traditional statistical survival techniques can achieve discriminative performance comparable to machine learning and deep learning without significant differences but are more applicable to practitioners with statistical backgrounds. IML models, such as AutoScore-Survival, can select the minimum number of variables and demonstrate competitive predictive performance, which is more easily understood and trusted by clinicians.

## Data Availability

All data created or analyzed during this investigation are included in this article and its supplementary information files. The code is available at https://github.com/nliulab/Survival-Benchmark.
