# Supplementary material for: Survival Modeling Using Deep Learning, Machine Learning, and Statistical Methods: A Comparative Analysis for Predicting Mortality After Hospital Admission
Source: Health Data Sci. 2026 Apr 20;6:0449. doi: 10.34133/hds.0449 (PMC13093892; doi:10.34133/hds.0449)
Supplement: Supplementary 1 — Appendix A to B Figs. S1 to S9 Tables S1 to S8 [file hds.0449.f1.docx]

**Appendix A**

Table S1. List of candidate variables and their abbreviations.

Table S2. Characteristics of study participants in SGH under different survival statues.

Table S3. Characteristics of study participants in MIMIC-IV under different survival statues.

Table S4. Description of various benchmark methods.

Table S5. Univariate and multivariate analysis of the Cox model in MIMIC-IV for survival probability.

Table S6. Sixteen-variable score in SGH for all-cause mortality for the inpatient dataset.

Table S7. Seven-variable score in MIMIC-IV for all-cause mortality for the inpatient dataset.

Table S8. Comparison of various models on secondary model attributes.

Figure S1. Kaplan-Meier curve of training and test cohort in MIMIC-IV.

Figure S2. Variable importance based on the AFT model.

Figure S3. Variable importance based on the CoxEN model.

Figure S4. Variable importance based on the stepwise CoxPH model.

Figure S5. Variable importance based on RSF.

Figure S6. Variable importance based on GBM.

Figure S7. SHAP variable importance based on DeepSurv.

Figure S8. SHAP variable importance based on CoxTime.

Figure S9. SHAP variable importance based on DeepHit.

**Table S1.** List of candidate variables and their abbreviations.

| Classification | Variables (Abbreviation) | Categorical/ Continuous |
| --- | --- | --- |
| Demographics  Information | Age | Continuous |
|  | Gender | Categorical |
|  | Race | Categorical |
| Vital signs | Triage class | Categorical |
|  | Diastolic blood pressure (Diastolic BP) | Continuous |
|  | Systolic blood pressure (Systolic BP) | Continuous |
|  | Fraction of inspiration oxygen (FIO2) | Categorical |
|  | Heart rate | Continuous |
|  | Respiratory rate | Continuous |
|  | Arterial oxygen saturation (SAO2) | Continuous |
|  | Temperature | Continuous |
| Laboratory results | Blood albumin (ALB) | Continuous |
|  | Basophils absolute count (BAS#) | Continuous |
|  | Basophils cell (BAS%) | Continuous |
|  | Bicarbonate (HCO3-) | Continuous |
|  | Chloride (Cl-) | Continuous |
|  | Serum creatinine (Cr) | Continuous |
|  | Eosinophils absolute count (EOS#) | Continuous |
|  | Eosinophils cell (EOS%) | Continuous |
|  | Blood glucose (GLU) | Continuous |
|  | Hematocrit (HCT) | Continuous |
|  | Hemoglobin (HGB) | Continuous |
|  | Lymphocytes absolute (LYMPH#) | Continuous |
|  | Lymphocytes cell (LYMPH%) | Continuous |
|  | Mean corpuscular hemoglobin (MCHB) | Continuous |
|  | Mean corpuscular hemoglobin concentration (MCHC) | Continuous |
|  | Mean corpuscular volume (MCV) | Continuous |
|  | Mean platelet volume (MPV) | Continuous |
|  | Monocytes absolute count (MONO#) | Continuous |
|  | Monocytes cell (MONO%) | Continuous |
|  | Neutrophils absolute count (NEUT#) | Continuous |
|  | Neutrophils cell (NEUT%) | Continuous |
|  | Platelet count (PLT) | Continuous |
|  | Potassium (K+) | Continuous |
|  | Red blood cell (RBC) | Continuous |
|  | Red cell distribution width (RDW) | Continuous |
|  | Serum sodium (Na+) | Continuous |
|  | Total absolute count (TAC) | Continuous |
|  | Total blood cells count (TCC) | Continuous |
|  | Troponin T quantitative (Troponin T) | Continuous |
|  | Blood urea nitrogen (BUN) | Continuous |
|  | White blood cell (WBC) | Continuous |
| Comorbidities | Myocardial infarction (MI) | Categorical |
|  | Congestive heart failure (CHF) | Categorical |
|  | Peripheral vascular diseases (PVD) | Categorical |
|  | Stroke | Categorical |
|  | Dementia | Categorical |
|  | Chronic pulmonary diseases (PulmonaryD) | Categorical |
|  | Rheumatic diseases (RheumaticD) | Categorical |
|  | Peptic ulcer disease (PUD) | Categorical |
|  | Hemiplegia or paraplegia (Paralysis) | Categorical |
|  | Renal diseases (Renal) | Categorical |
|  | Malignancy | Categorical |
|  | Liver diseases (LiverD) | Categorical |
|  | Diabetes | Categorical |
| History information | Emergency admissions in the past year (ED#) | Continuous |
|  | Inpatient admission in the past year (INP#) | Continuous |
|  | Surgeries in the past year (SURG#) | Continuous |
|  | HUD admission in the past year (HD#) | Continuous |
|  | ICU admission in the past year (ICU#) | Continuous |

**Table S2.** Characteristics of study participants in SGH under different survival statues.

|  | Status | |  |
| --- | --- | --- | --- |
| Characteristic | **False** | **Ture** | ***P*-value** |
| No. of participants | 112,118 (89.8%) | 12,755 (10.2%) |  |
| Age (years) | 67.00 [56.00, 78.00] | 74.00 [64.00, 83.00] | <0.001 |
| Gender |  |  | <0.001 |
| Male | 54,887 (49.0%) | 6,958 (54.6%) |  |
| Female | 57,231 (51.0%) | 5,797 (45.4%) |  |
| Race |  |  | <0.001 |
| Chinese | 82,003 (73.1%) | 10,357 (81.2%) |  |
| Indian | 12,001 (10.7%) | 845 (6.6%) |  |
| Malay | 13,436 (12.0%) | 1,232 (9.7%) |  |
| Others | 4,668 (4.2%) | 321 (2.5%) |  |
| Triage class |  |  | <0.001 |
| P1 | 23,766 (21.2%) | 4,864 (38.1%) |  |
| P2 | 72,822 (65.0%) | 7,371 (57.8%) |  |
| P3 & P4 | 15,475 (13.8%) | 520 (4.1%) |  |
| Diastolic blood pressure (mmHg) | 72.00 [63.00, 81.00] | 67.00 [58.00, 76.00] | <0.001 |
| Systolic blood pressure (mmHg) | 135.00 [119.00, 154.00] | 124.00 [107.00, 141.00] | <0.001 |
| FIO2 |  |  | <0.001 |
| >21 | 111,731 (99.7%) | 12,584 (98.7%) |  |
| =21 | 387 (0.3%) | 171 (1.3%) |  |
| Pulse (bpm) | 83.00 [72.00, 96.00] | 91.00 [78.00, 105.00] | <0.001 |
| Respiratory rate (cpm) | 18.00 [17.00, 19.00] | 18.00 [18.00, 20.00] | <0.001 |
| SAO2 (%) | 98.00 [97.00, 99.00] | 98.00 [96.00, 99.00] | <0.001 |
| Temperature (℃) | 36.60 [36.10, 37.10] | 36.50 [36.10, 37.10] | <0.001 |
| Blood albumin (g/L) | 39.00 [39.00, 39.00] | 39.00 [31.00, 39.00] | <0.001 |
| Basophils absolute ($\boldsymbol{10}^{\boldsymbol{9}}$/L) | 0.04 [0.03, 0.05] | 0.04 [0.02, 0.05] | <0.001 |
| Basophil cell (%) | 0.40 [0.30, 0.60] | 0.40 [0.20, 0.50] | <0.001 |
| Bicarbonate (mmol/L) | 23.40 [21.40, 25.30] | 23.10 [19.90, 25.40] | <0.001 |
| Chloride (mmol/L) | 103.00 [100.00, 105.00] | 100.00 [95.00, 103.00] | <0.001 |
| Serum creatinine ($\boldsymbol{\mu}$mol/L) | 77.00 [63.00, 114.00] | 88.00 [63.00, 168.00] | <0.001 |
| Eosinophils absolute ($\boldsymbol{10}^{\boldsymbol{9}}$/L) | 0.11 [0.04, 0.21] | 0.06 [0.01, 0.14] | <0.001 |
| Eosinophil cell (%) | 1.40 [0.50, 2.60] | 0.70 [0.10, 1.60] | <0.001 |
| Blood glucose (mmol/L) | 6.50 [5.90, 8.80] | 6.80 [6.10, 9.10] | <0.001 |
| Hematocrit (%) | 38.10 [33.50, 41.00] | 33.00 [27.90, 38.30] | <0.001 |
| Hemoglobin (g/dL) | 12.70 [11.00, 13.70] | 10.80 [9.10, 12.60] | <0.001 |
| Lymph absolute ($\boldsymbol{10}^{\boldsymbol{9}}$/L) | 1.59 [1.06, 2.05] | 1.10 [0.70, 1.65] | <0.001 |
| Lymph cell (%) | 19.20 [11.60, 25.20] | 11.50 [6.60, 19.20] | <0.001 |
| MCHB (pg/g) | 29.80 [28.20, 30.80] | 29.80 [27.80, 31.10] | <0.001 |
| MCHC (g/L) | 33.30 [32.40, 34.00] | 32.90 [31.80, 33.70] | <0.001 |
| Mean corpuscular volume (fL) | 88.40 [84.90, 92.00] | 89.10 [85.20, 94.30] | <0.001 |
| Mean platelet volume (fL) | 9.90 [9.40, 10.40] | 9.90 [9.30, 10.60] | **0.030** |
| Monocytes absolute ($\boldsymbol{10}^{\boldsymbol{9}}$/L) | 0.62 [0.50, 0.84] | 0.67 [0.50, 0.96] | <0.001 |
| Monocytes cell (%) | 7.30 [6.00, 9.10] | 7.30 [5.30, 9.20] | <0.001 |
| Neutrophils absolute count ($\boldsymbol{10}^{\boldsymbol{9}}$/L) | 5.70 [4.41, 8.47] | 7.29 [5.26, 11.06] | <0.001 |
| Neutrophils cell (%) | 69.20 [62.30, 79.10] | 78.10 [68.40, 85.90] | <0.001 |
| Platelet count ($\boldsymbol{10}^{\boldsymbol{9}}$/L) | 248.00 [204.00, 308.00] | 248.00 [175.00, 335.00] | <0.001 |
| Serum potassium (mmol/L) | 4.00 [3.80, 4.40] | 4.10 [3.80, 4.60] | <0.001 |
| Red blood cell ($\boldsymbol{10}^{\boldsymbol{12}}$/L) | 4.36 [3.78, 4.73] | 3.70 [3.11, 4.34] | <0.001 |
| Red cell distribution width (%) | 13.40 [12.90, 14.60] | 15.20 [13.70, 17.40] | <0.001 |
| Serum sodium (mmol/L) | 137.00 [135.00, 139.00] | 135.00 [131.00, 138.00] | <0.001 |
| Total absolute count ($\mathbf{10}^{\mathbf{9}}$/L) | 8.58 [6.93, 11.30] | 9.55 [7.34, 13.56] | <0.001 |
| Total blood cells count ($\boldsymbol{10}^{\boldsymbol{9}}$/L) | 100.00 [100.00, 100.00] | 100.00 [100.00, 100.00] | **0.032** |
| Troponin T quantitative (ng/mL) | 0.14 [0.14, 13.00] | 0.14 [0.14, 27.00] | <0.001 |
| Blood urea nitrogen (mmol/L) | 5.50 [4.30, 8.60] | 7.90 [4.90, 14.00] | <0.001 |
| White blood cell ($\boldsymbol{10}^{\boldsymbol{9}}$/L) | 8.58 [6.91, 11.30] | 9.55 [7.29, 13.56] | <0.001 |
| MI | 4,600 (4.1%) | 1,482 (11.6%) | <0.001 |
| CHF | 7,367 (6.6%) | 1,497 (11.7%) | <0.001 |
| PVD | 42,41 (3.8%) | 841 (6.6%) | <0.001 |
| Stroke | 11,064 (9.9%) | 1,408 (11.0%) | <0.001 |
| Dementia | 3,473 (3.1%) | 732 (5.7%) | <0.001 |
| Pulmonary | 7,998 (7.1%) | 1,091 (8.6%) | <0.001 |
| Rheumatic | 1,394 (1.2%) | 123 (1.0%) | 0.007 |
| PUD | 2,044 (1.8%) | 389 (3.0%) | <0.001 |
| Paralysis | 4,160 (3.7%) | 545 (4.3%) | 0.002 |
| Renal | 24,469 (21.8%) | 3,871 (30.3%) | <0.001 |
| Malignancy |  |  | <0.001 |
| None | 98,530 (87.9%) | 5,965 (46.8%) |  |
| Local tumor leukemia and lymphoma | 7,838(7.0%) | 1,724 (13.5%) |  |
| Metastatic solid tumor | 5,750 (5.1%) | 5,066 (39.7%) |  |
| Liver disease |  |  | <0.001 |
| None | 107,104(95.5%) | 11,667 (91.5%) |  |
| Mild | 3,614 (3.2%) | 593 (4.6%) |  |
| Severe | 1,400 (1.2%) | 495 (3.9%) |  |
| Diabetes |  |  | <0.001 |
| None | 72,322 (64.5%) | 7,618 (59.7%) |  |
| Diabetes without chronic complications | 3,439 (3.2%) | 331 (2.6%) |  |
| Diabetes with complications | 36,357 (32.4%) | 4,806 (37.7%) |  |

*Continuous variables are presented as Median [IQR]; binary/categorical variables are presented as Count (%).

**Table S3.** Characteristics of study participants in MIMIC-IV under different survival statues.

|  | Status | |  |  |
| --- | --- | --- | --- | --- |
| Characteristic | **False** | **Ture** | **Missing rate** | ***P*-value** |
| No. of participants | 6769 (98.3%) | 120 (1.7%) |  |  |
| Age (years) | 61.00 [43.00, 75.00] | 72.00 [58.00, 83.25] | - | <0.001 |
| Gender |  |  | - | <0.001 |
| Male | 3194 (47.2%) | 77 (64.2%) |  |  |
| Female | 3695 (52.8%) | 43 (35.8%) |  |  |
| Triage class |  |  | 0.03% | <0.001 |
| ESI1 | 435 (6.4%) | 31 (25.8%) |  |  |
| ESI2 | 3382 (50.0%) | 66 (55.0%) |  |  |
| ESI3 & ESI4 & ESI5 | 2952 (43.6%) | 23 (19.2%) |  |  |
| Diastolic blood pressure (mmHg) | 75.00 [65.00, 84.00] | 70.00 [60.00, 80.00] | - | 0.001 |
| Systolic blood pressure (mmHg) | 130.00 [116.00, 148.00] | 118.00 [108.00, 140.00] | - | <0.001 |
| Pulse (bpm) | 84.00 [72.00, 99.00] | 97.50 [80.00, 110.00] | 0.09% | <0.001 |
| Respiratory rate (cpm) | 18.00 [16.00, 18.00] | 18.00 [16.00, 20.00] | 0.9% | 0.008 |
| SAO2 (%) | 99.00 [97.00, 100.00] | 98.00 [95.00, 100.00] | - | <0.001 |
| Temperature (F) | 98.20 [97.60, 98.80] | 98.30 [97.60, 99.15] | 1.5% | 0.313 |
| MI | 278 (4.1%) | 8 (6.7%) | - | 0.245 |
| CHF | 683 (10.1%) | 23 (19.2%) | - | 0.002 |
| PVD | 315 (4.7%) | 8 (6.7%) | - | 0.414 |
| Stroke | 384 (5.7%) | 16 (13.3%) | - | 0.001 |
| Dementia | 141 (2.1%) | 7 (5.8%) | - | 0.013 |
| Pulmonary | 768 (11.3%) | 24 (20.0%) | - | 0.005 |
| Rheumatic | 139 (2.1%) | 3 (2.5%) | - | 0.986 |
| PUD | 185 (2.7%) | 5 (4.2%) | - | 0.503 |
| Paralysis | 101 (1.5%) | 3 (2.5%) | - | 0.603 |
| Renal | 900 (13.3%) | 25 (20.8%) | - | 0.023 |
| Malignancy |  |  | - | <0.001 |
| None | 5760 (85.1%) | 41 (34.2%) |  |  |
| Local tumor leukemia and lymphoma | 847 (12.5%) | 49 (40.8%) |  |  |
| Metastatic solid tumor | 162 (2.4%) | 30 (25.0%) |  |  |
| Liver disease |  |  | - | <0.001 |
| None | 6022 (88.9%) | 88 (73.3%) |  |  |
| Mild | 586 (8.7%) | 24 (20.0%) |  |  |
| Severe | 161 (2.4%) | 8 (6.7%) |  |  |
| Diabetes |  |  | - | 0.342 |
| None | 5075 (75.0%) | 87 (72.5%) |  |  |
| Diabetes without chronic complications | 1158 (17.1%) | 25 (20.8%) |  |  |
| Diabetes with complications | 536 (7.9%) | 8 (6.7%) |  |  |

*Continuous variables are presented as Median [IQR]; binary/categorical variables are presented as Count (%).

**Table S4.** Description of various benchmark methods

| Models | Description | Hyperparameters tuning set | Software (Package) |
| --- | --- | --- | --- |
| Accelerated failure time model (AFT) | Traditional statistical method | None | R (rms) |
| Cox proportional hazards model (CoxPH) | Traditional statistical method | None | R (survival) |
| Stepwise CoxPH | Traditional statistical method | None | Python (lifelines) |
| Cox model with elastic net penalty (CoxEN) | Traditional statistical method | Penalty = ‘Elastic net’,  Alpha = [0, ${10}^{-2}$],  The EN mixing parameter l1 ratio= 0.9,  Minimum alpha ratio = 0.01 | Python (scikit-surv) |
| AutoScore-Survival | Interpretability machine learning | Number of trees = {500, 1000, 1500},  Number of variables tuned through performance-based parsimony plot. | R (AutoScore) |
| Random survival forest (RSF) | Ensemble machine learning | Number of trees = {500, 1000, 1500},  Number of variables to split at each node = UniformInt[2, 4],  Number of random splits = UniformInt[3, 10],  Minimum size of the terminal node = {3, 5, 10, 15}. | R (randomForestSRC) |
| Gradient Boosting (GBM) | Ensemble machine learning | Number of trees = {500, 1000, 1500},  Learning rate = Uniform[1e-5, 1],  Subsample = Uniform[0.5, 1],  Minimum number of samples required to perform the spits =[2, 10],  Minimum number of sample at the leaf node = UniformInt[1, 3],  Maximum depth = UniformInt[3, 10]. | Python (scikit-surv) |
| DeepSurv | Feedforward deep neural network | Activation = “relu”,  Drop out = [0.1, 0.4],  Learning rate = {0.01, 0.005, 0.001},  Batch size = {256, 512},  Epoch = 64,  Loss = partial log-likelihood,  Early stopping = True,  Optimizer = “Adam”,  Notes = {[64, 64, 32], [128, 64, 32], [128, 64, 64, 32]}. | Python (Pycox) |
| CoxTime | Feedforward deep neural network | Activation = “relu”,  Drop out = [0.1, 0.4],  Learning rate = {0.01, 0.005, 0.001},  Batch size = {256, 512},  Epoch = 64,  Loss = partial log-likelihood,  Early stopping = True,  Optimizer = “Adam”,  Notes = {[64, 64, 32], [128, 64, 32], [128, 64, 64, 32]}. | Python (Pycox) |
| DeepHit | Feedforward deep neural network | Activation = “relu”,  Drop out = [0.1, 0.4],  Learning rate = {0.01, 0.005, 0.001},  Batch size = {256, 512},  Epoch = 64,  Loss = partial log-likelihood,  Early stopping = True,  Optimizer = “Adam”,  Notes = {[64, 64, 32], [128, 64, 32], [128, 64, 64, 32]}. | Python (Pycox) |

*Braces are meant to be sets and brackets are meant to ranges.

**Table S5**: Univariate and multivariate analysis of the Cox model in MIMIC-IV for survival probability.

| Variables | Univariate Cox Regression | | Multivariate Cox Regression | |
| --- | --- | --- | --- | --- |
|  | Hazard Ratio (95%) | P-value | Hazard Ratio (95%) | P-value |
| Age (years) | 1.026 (1.014 – 1.038) | < 0.001 | 1.025 (1.010 – 1.040) | 0.001 |
| Gender (Male) | 1.447 (0.994 – 2.105) | 0.0538 | 1.393 (0.935 – 2.075) | 0.1033 |
| Triage class |  |  |  |  |
| ESI1 | - | - | - | - |
| ESI2 | 0.429 (0.279 – 0.659) | 0.001 | 0.506 (0.313 – 0.820) | 0.0057 |
| ESI3&4&5 | 0.223 (0.129 – 0.383) | <0.001 | 0.283 (0.154 – 0.518) | <0.001 |
| Diastolic BP | 0.988 (0.977 – 1.000) | 0.0559 | 1.000 (0.984 – 1.015) | 0.9816 |
| Systolic BP | 0.995 (0.988 – 1.003) | 0.1957 | 0.997 (0.988 – 1.007) | 0.5655 |
| Heart rate | 1.011 (1.002 – 1.019) | 0.0115 | 1.006 (0.997 – 1.016) | 0.1982 |
| Respiration rate | 1.036 (0.994 – 1.080) | 0.0933 | 0.980 (0.936 – 1.026) | 0.3844 |
| SAO2 | 0.957 (0.940 – 0.975) | <0.001 | 0.963 (0.935 – 0.992) | 0.0121 |
| Temperature | 0.967 (0.941 – 0.995) | 0.0204 | 0.962 (0.937 – 0.988) | 0.0045 |
| MI | 1.243 (0.606 – 2.550) | 0.5522 | 1.179 (0.502 – 2.770) | 0.7049 |
| CHF | 1.446 (0.917 – 2.280) | 0.1128 | 1.348 (0.731 – 2.486) | 0.3381 |
| PVD | 1.080 (0.526 – 2.216) | 0.8339 | 0.847 (0.389 – 1.843) | 0.6756 |
| Stroke | 1.785 (1.054 – 3.025) | 0.0312 | 1.610 (0.843– 3.077) | 0.1494 |
| Dementia | 2.647 (1.231 – 5.692) | 0.0127 | 1.962 (0.837 – 4.603) | 0.1212 |
| Pulmonary | 1.478 (0.944 – 2.314) | 0.0878 | 0.945 (0.555 – 1.611) | 0.8367 |
| Rheumatic | 1.031 (0.328 – 3.247) | 0.9580 | 1.115 (0.337 – 3.694) | 0.8580 |
| PUD | 0.902 (0.368 – 2.211) | 0.8215 | 0.610 (0.237 – 1.566) | 0.3039 |
| Paralysis | 1.103 (0.350 – 3.475) | 0.8670 | 0.560 (0.156 – 2.004) | 0.3726 |
| Renal | 1.289 (0.828 – 2.005) | 0.2613 | 1.260 (0.723 – 2.194) | 0.4149 |
| Malignancy |  |  |  |  |
| None | - |  |  |  |
| Local tumor leukemia and lymphoma | 2.620 (1.817 – 3.778) | <0.001 | 2.131 (1.211 – 3.752) | 0.0087 |
| Metastatic solid tumor | 2.917 (1.923 – 4.424) | <0.001 | 1.788 (0.979 – 3.266) | 0.0588 |
| LiverD |  |  |  |  |
| None | - |  |  |  |
| Mild | 1.516 (0.967 – 2.376) | 0.0698 | 1.359 (0.775 – 2.385) | 02843 |
| Severe | 1.762 (0.858 – 3.618) | 0.1226 | 1.811 (0.748 – 4.382) | 0.1878 |
| Diabetes |  |  |  |  |
| None | - |  |  |  |
| Diabetes without chronic complications | 0.934 (0601 – 1.454) | 0.7637 | 0.618 (0.370 – 1.033) | 00663 |
| Diabetes with complications | 0.651 (0.318 – 1.336) | 0.2420 | 0.495 (0.200 – 1.225) | 0.1283 |
| ED# | 1.113 (1.024 – 1.210) | 0.0115 | 1.181 (0.981 – 1.421) | 0.0781 |
| INP# | 1.053 (0.985 – 1.125) | 0.1329 | 0.873 (0.738 – 1.031) | 0.1100 |
| ICU# | 1.030 (0.851 – 1.246) | 0.7634 | 0.936 (0.708 – 1.237) | 0.6420 |

**Table S6.** Sixteen-variable score in SGH for all-cause mortality for the inpatient dataset.

| Variables | Interval | Point |
| --- | --- | --- |
| Malignancy | NA | **0** |
|  | 1local | **7** |
|  | 2metastatic | **15** |
| Total cell count (TCC) | < 100 | **0** |
|  | $\geq100$ | **6** |
| Age | [21, 41) | **0** |
|  | [41, 58) | **7** |
|  | [58, 76) | **10** |
|  | [76, 85) | **13** |
|  | $\geq$85 | **17** |
| Respiratory rate | <16 | **2** |
|  | [16, 17) | **0** |
|  | [17, 18) | **1** |
|  | [18, 20) | **1** |
|  | $\geq$ 20 | **3** |
| Diastolic BP | < 79 | **0** |
|  | [79, 91) | **1** |
|  | $\geq91$ | **2** |
| Blood albumin (ALB) | < 34 | **10** |
|  | [34, 39) | **5** |
|  | [39, 41) | **6** |
|  | $\geq$41 | **0** |
| SAO2 | < 95 | **4** |
|  | [95, 97) | **1** |
|  | $\geq$ 97 | **0** |
| Heart rate | < 75 | **0** |
|  | [75, 94) | **1** |
|  | [94, 109) | **2** |
|  | $\geq109$ | **4** |
| Troponin T Quantitative | < 13 | **2** |
|  | [13, 36) | **0** |
|  | $\geq$ 36 | **4** |
| Blood urea nitrogen (BUN) | < 4.7 | **0** |
|  | [4.7, 8) | **2** |
|  | [8, 16.4) | **4** |
|  | $\geq$ 16.4 | **6** |
| Systolic BP | < 105 | **6** |
|  | [105, 121) | **5** |
|  | [121, 148) | **4** |
|  | [148, 174) | **2** |
|  | $\geq174$ | **0** |
| Sodium | < 95 | **8** |
|  | [95, 100) | **5** |
|  | [100, 104) | **3** |
|  | [104, 107) | **0** |
|  | $\geq$ 107 | **1** |
| Bicarbonate | < 18.8 | **3** |
|  | [18.8, 27.2) | **0** |
|  | $\geq$ 27.2 | **2** |
| Chloride | <95 | **8** |
|  | [95, 100) | **5** |
|  | [100, 104) | **3** |
|  | [104, 107) | **0** |
|  | $\geq$ 107 | **1** |
| BAS# | < 0.02 | **2** |
|  | [0.02, 0.03) | **1** |
|  | [0.03, 0.05) | **1** |
|  | [0.05, 0.07) | **0** |
|  | $\geq$ 0.07 | **1** |
| RDW | < 12.3 | **0** |
|  | [12.3, 13.1) | **1** |
|  | [13.1, 14.6) | **4** |
|  | [14.6, 17.2) | **7** |
|  | $\geq$17.2 | **10** |

**Table S7.** Seven-variable score in MIMIC-IV for all-cause mortality for the inpatient dataset.

| Variables | Interval | Point |
| --- | --- | --- |
| AGE | [21, 27) | **0** |
|  | [27, 38) | **2** |
|  | [38, 79) | **5** |
|  | [79, 85) | **12** |
|  | $\geq$85 | **21** |
| SAO2 | < 96 | **12** |
|  | [96, 97) | **4** |
|  | $\geq$ 97 | **0** |
| Triage | ESI1 | **23** |
|  | ESI2 | **13** |
|  | ESI3 & 4 & 5 | **0** |
| Malignancy | NA & local | **0** |
|  | 2metastatic | **19** |
| Respiratory rate | <16 | **18** |
|  | [16, 18) | **0** |
|  | [18, 20) | **1** |
|  | $\geq$ 20 | **6** |
| ICU# | <1 | **2** |
|  | $\geq1$ | **0** |
| ED# | < 1 | **0** |
|  | [1, 3) | **5** |
|  | $\geq$ 3 | **4** |

Table S8. Comparison of various models on secondary model attributes.

| Classification | Models | Variable select | Model file size in SGH cohort | Model file size in MIMIC-IV cohort |
| --- | --- | --- | --- | --- |
| Traditional statistical method | AFT model | No | 12KB | 12KBKB |
|  | CoxPH model | No | 13KB | 10KB |
|  | Stepwise CoxPH | Yes | 99KB | 79KB |
|  | CoxEN | Yes | 166KB | 147KB |
| Ensemble machine learning | RSF | Yes | 11KB | 11KB |
|  | GBM | Yes | 180KB | 127KB |
| Interpretability machine learning | AutoScore-Survival | Yes | 14KB | 14KB |
| Feedforward deep neural network | DeepSurv | No | 258KB | 164KB |
|  | CoxTime | No | 269KB | 162KB |
|  | DeepHit | No | 333KB | 252KB |

**Figure S1.** Kaplan-Meier curve of training and test cohort in MIMIC-IV.


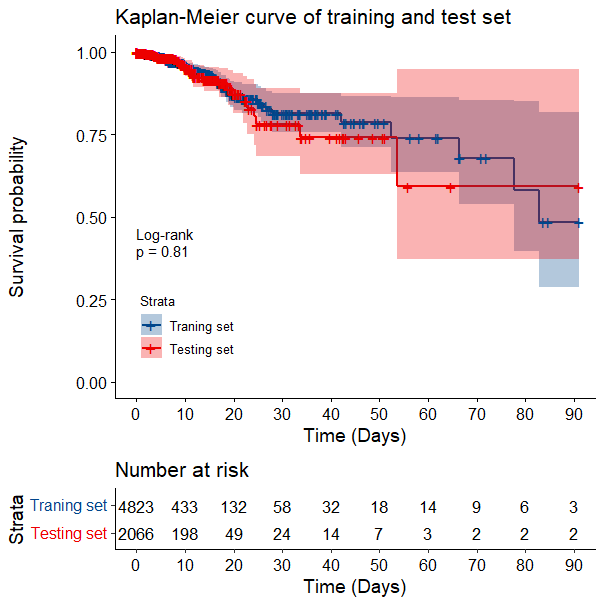


**Figure S2.** Variable importance based on the AFT model.


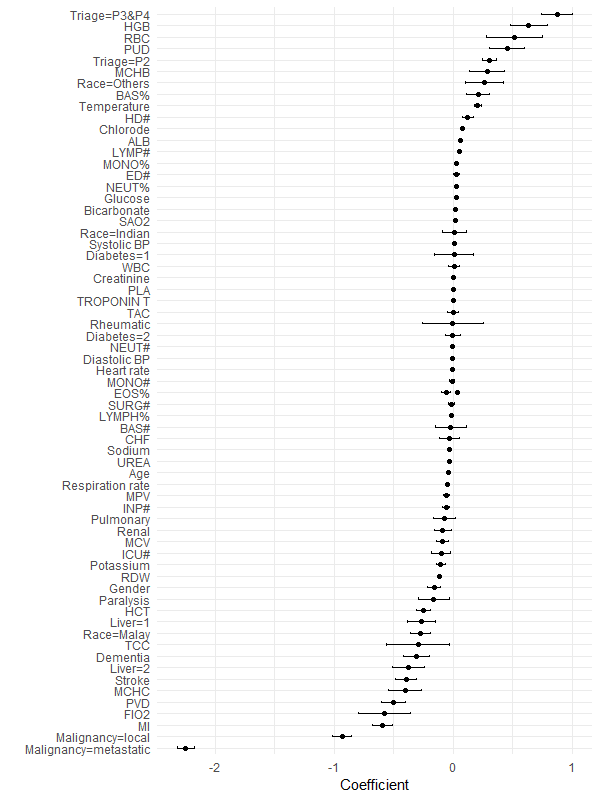


**Figure S3.** Variable importance based on the CoxEN model.


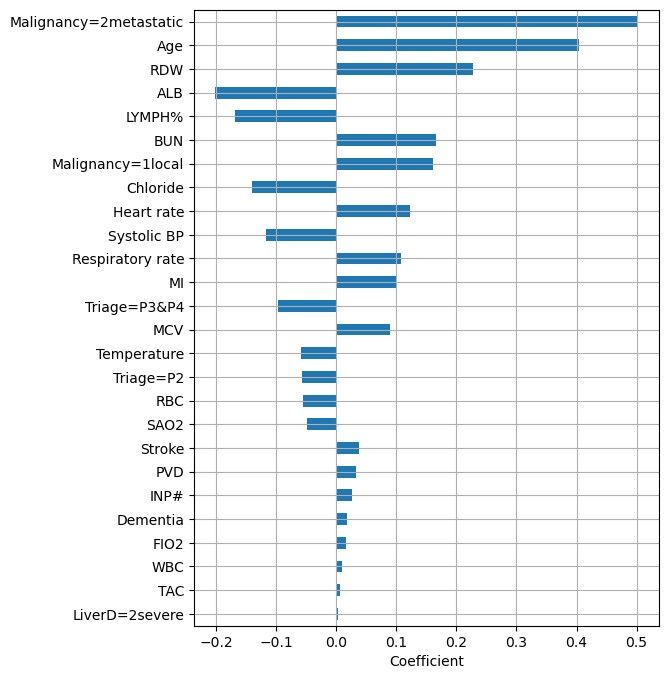


**Figure S4.** Variable importance based on the stepwise CoxPH method.


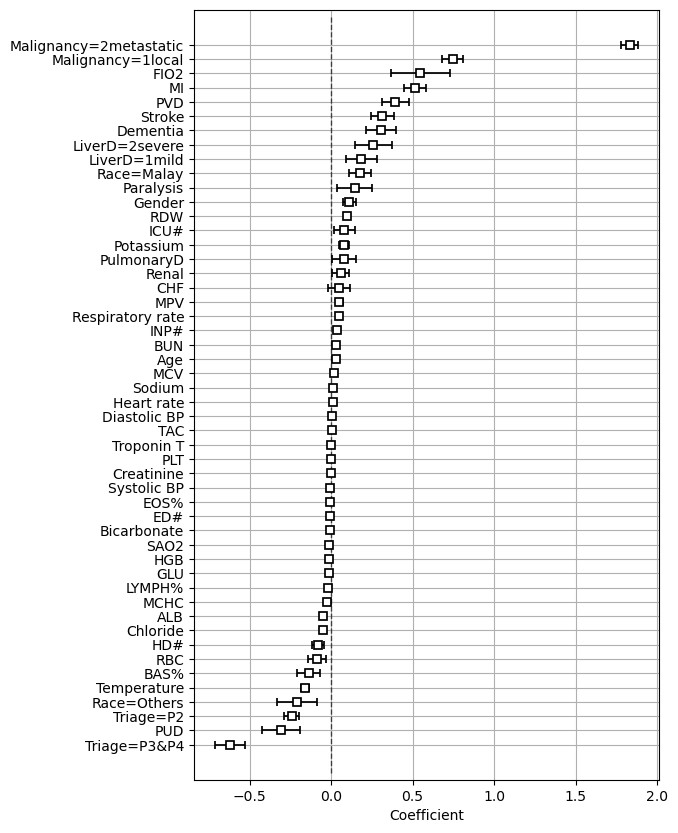


**Figure S5.** Variable importance based on RSF.


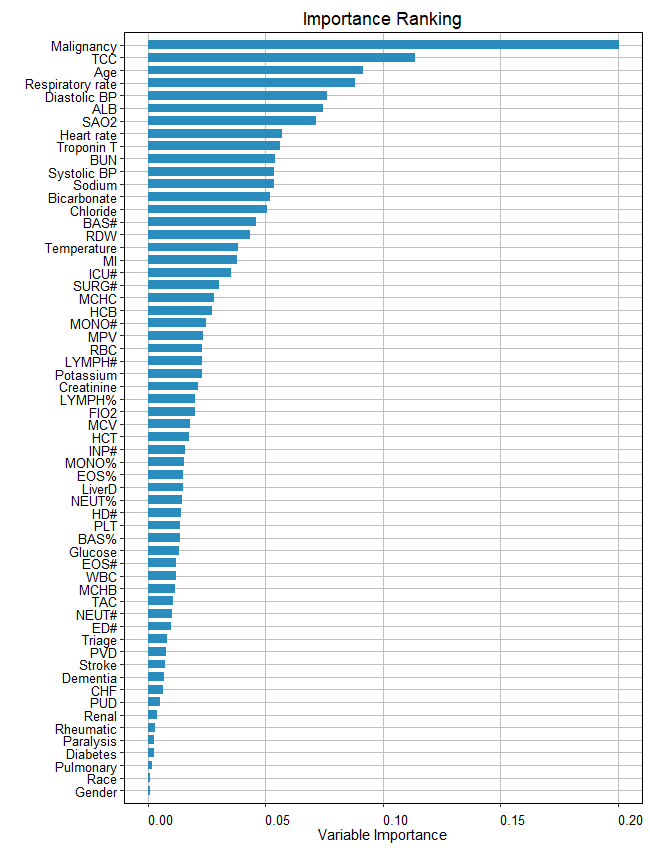


**Figure S6.** Variable importance based on GBM.


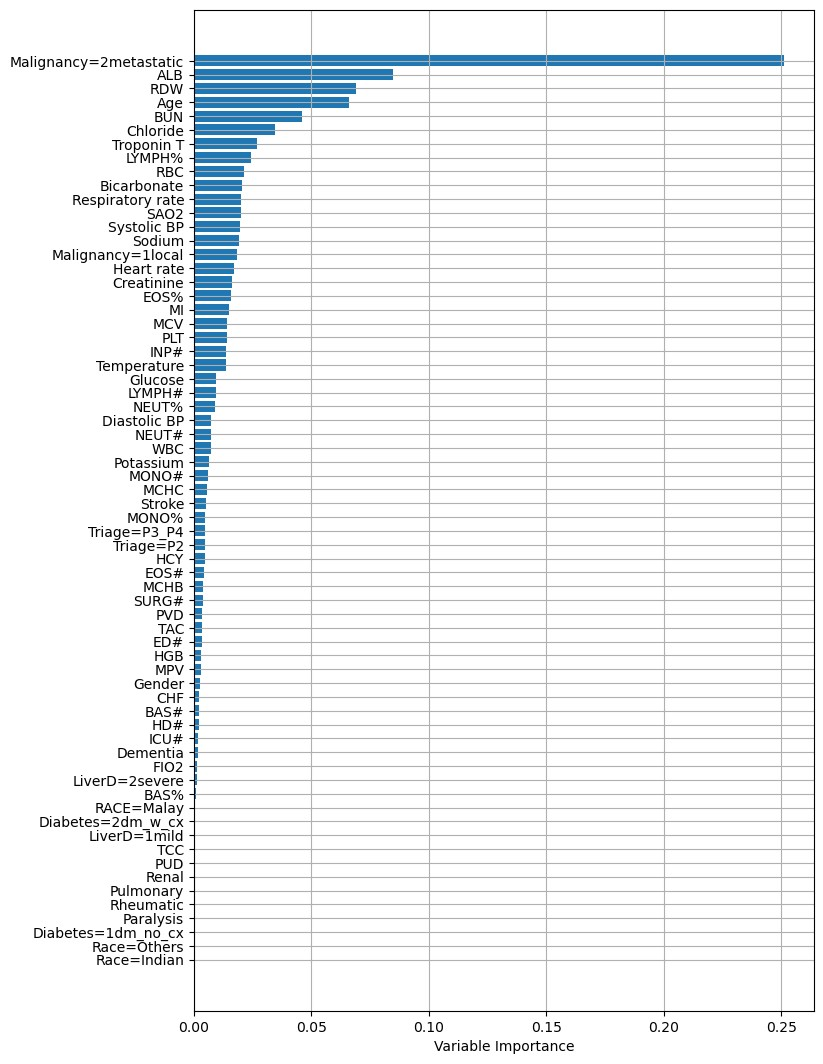


**Figure S7.** SHAP variable importance based on DeepSurv.


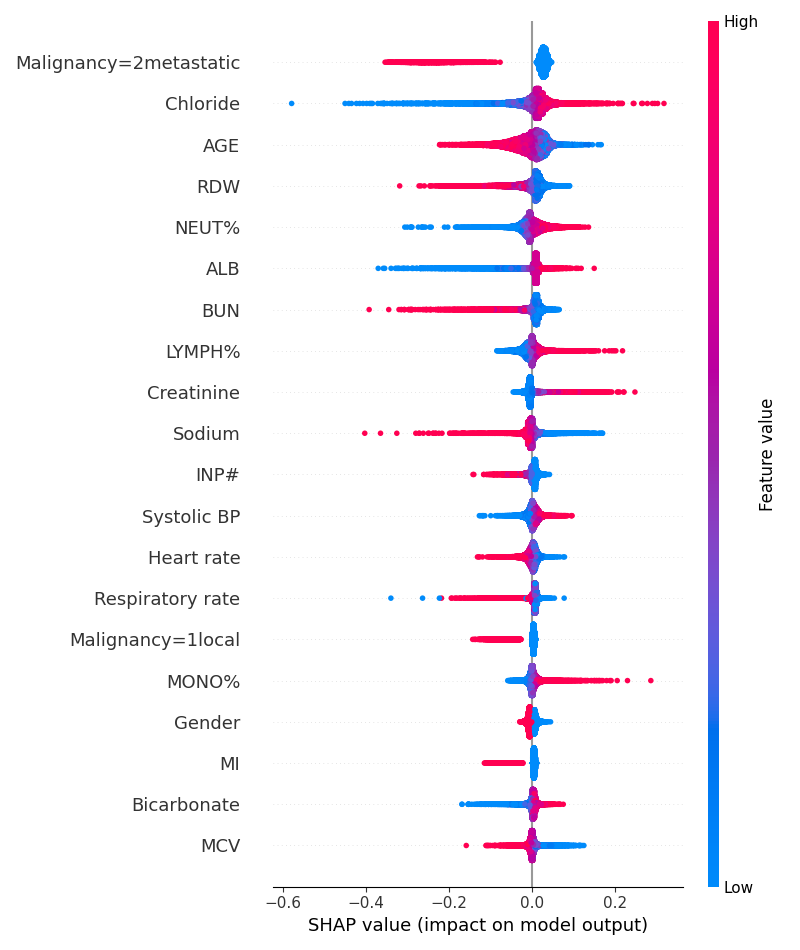


**Figure S8.** SHAP variable importance based on CoxTime.


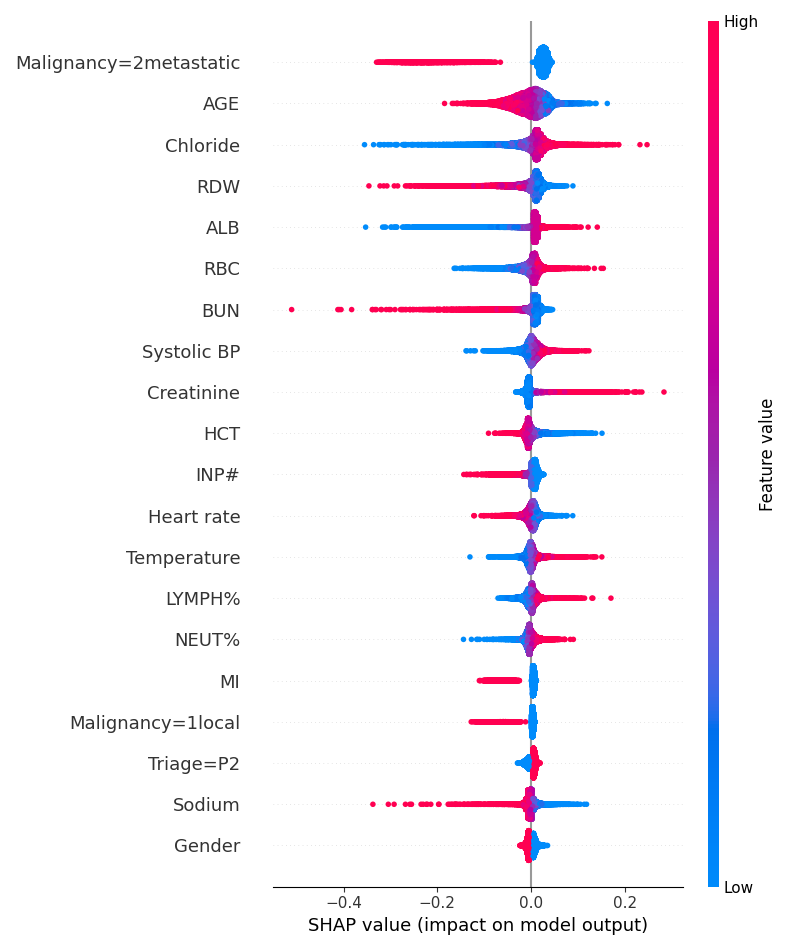


**Figure S9.** SHAP variable importance based on DeepHit.


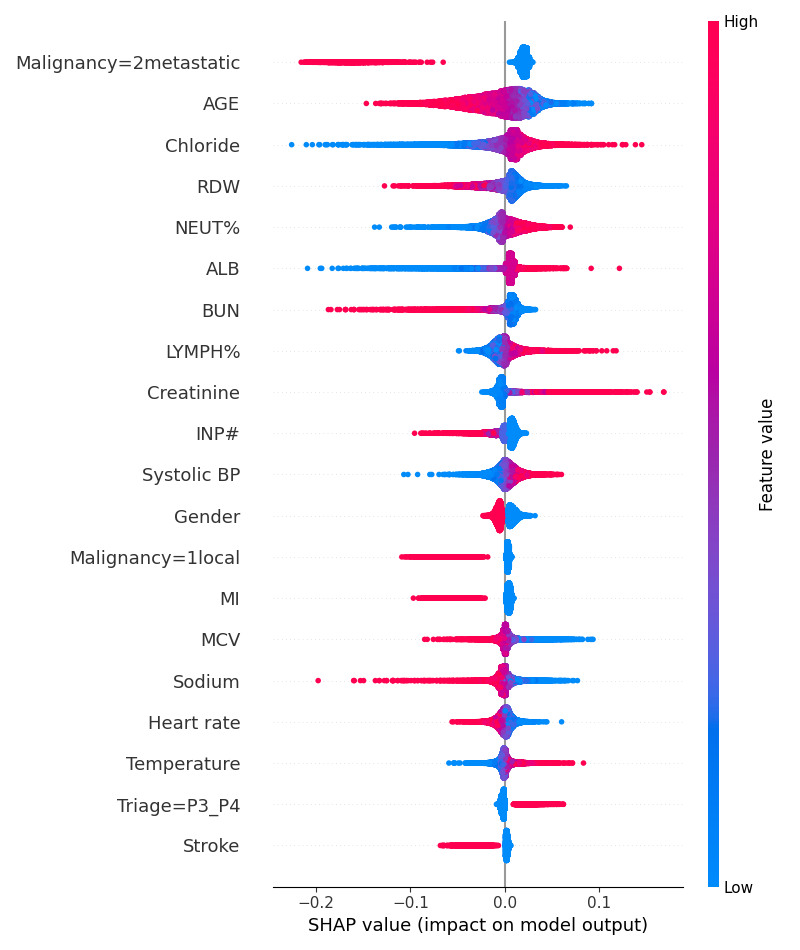


**Appendix B**

Let R denote the risk score, without loss of generality, suppose that larger values of the risk score R are associated with greater hazards. The C-index can be defined as the proportion of concordance pairs among the population, $P(R_{i}>R_{j}|T_{i}<T_{j})$, where ($R_{i}, T_{i}$) and ($R_{j}, T_{j}$) indicate two independent observations from two randomly chosen subjects $i$ and $j$. The empirical C-index can be defined as

$$\hat{C}=\frac{\sum_{i<j} \delta_{i}I(Y_{i}<Y_{j})I(\hat{R}_{i}>\hat{R}_{j})+\delta_{j}I(Y_{j}<Y_{i})I(\hat{R}_{j}>\hat{R}_{i})}{\sum_{i<j} \delta_{i}I(Y_{i}<Y_{j})+\delta_{j}I(Y_{j}<Y_{i})}.$$

The BS for survival data for a cohort І is define as

$$BS \left( t,\hat{S} \right)=N^{-1}\sum_{i\inІ} \frac{1-I\left( Y_{j}<t,\delta_{i}=0 \right)}{\hat{G}\left( Y_{i}\wedge t \right)}\{{I\left( Y_{j}>t \right)-\hat{S}\left( t | X_{i} \right)\}}^{2},$$

where $\hat{G}\left( \cdot\right)$ is the Kaplan-Meier estimator of the survival function for the censoring times, and

$I(\cdot)$ denotes an indication function. Then the IBS can be obtained by calculating the average of BSs over all observed time points $\left( 0, \tau\right)$ , which is given by IBS $= \tau^{-1}\int_{0}^{\tau} BS(u,\hat{S})du$.
